# Review of the genus *Coccus* Linnaeus from Korea, with description of a new species (Hemiptera, Coccomorpha, Coccidae)

**DOI:** 10.3897/zookeys.734.22774

**Published:** 2018-02-05

**Authors:** Jinyeong Choi, Seunghwan Lee

**Affiliations:** 1 Insect Biosystematics Laboratory, Department of Agricultural Biotechnology, Seoul National University, Seoul 151-921, Republic of Korea; 2 Research Institute of Agriculture and Life Sciences, Seoul National University, Seoul 151-921, Republic of Korea

**Keywords:** Coccinae, Coccini, soft scale insect, taxonomy

## Abstract

The genus *Coccus* from Korea is reviewed, including a new species, *C. ficicola* **sp. n.**, and a first record of *C. malloti* (Takahashi, 1956). The new species is characterized by a distinctive pattern of ventral tubular ducts on medial area of head and thorax, and submarginal area of abdomen. The adult female of *Coccus ficicola* **sp. n.** is described and illustrated, and a morphological comparison is given with congeners. *Coccus malloti* is redescribed and illustrated based on the adult female specimens from Korea. A key to the four species of *Coccus* known from Korea is provided with diagnoses and photographs.

## Introduction

The genus *Coccus* Linnaeus, 1758, which is a species-rich group in the family Coccidae, comprises approximately 111 species worldwide (Hodgson 1994; García-Morales et al. 2016). This group is defined morphologically by the distribution of ventral tubular ducts, the shape of dorsal and marginal setae, and the presence of a tibio-tarsal articulatory sclerosis on each leg (Hodgson 1994); however, the molecular phylogeny using some of the taxa included in Coccidae revealed that it is not a monophyletic group and needs taxonomic revision (Lin et al. 2013). Among the genus *Coccus*, *C. hesperidum* Linnaeus, *C. viridis* Green, and *C. celatus* De Lotto are known as economic pests of ornamental and agricultural products (Kapranas et al. 2007; Waller et al. 2007). Two species of the genus, *C. hesperidum* and *C. pseudomagnoliarum* (Kuwana) have been previously recorded from Korea. Here, a new species is described, *Coccus ficicola* sp. n. and its morphology is compared with congeners. A hitherto unrecorded species is also redescribed, *C. malloti* (Takahashi) and recorded for the first time from Korea.

## Materials and methods

The specimens were mounted on microscope slides using the method of Hodgson and Henderson (2000) and Danzig and Gavrilov-Zimin (2014). The micrographs of slide-mounted materials were taken and measured using analysis software (Active Measure ver. 3.0.3, Mitani Co. Ltd, Japan). The terminology follows Hodgson (1994) and Hodgson and Henderson (2000), except that the term “pregenital disc-pores” is replaced with “multilocular pores” suggested by Kondo and Hardy (2008). The type specimens are deposited in the Insect Biosystematics Laboratory, Research Institute for Agriculture and Life Science, Seoul National University, Korea (**SNU**).

## Taxonomy

### Genus Coccus

Taxon classificationAnimaliaHemipteraCoccidae

Linnaeus, 1758: 455

#### Type species.

*Coccus hesperidum* Linnaeus, 1758, designated by Opinion 1303 (1985).

#### Diagnosis.

Dorsal setae pointed or blunt; dorsal tubular ducts and dorsal tubercles present or absent; marginal setae with pointed or frayed apices; ventral tubular ducts present or absent, if present, mainly distributed on medial area of thorax or submarginal area; a tibio-tarsal articulatory sclerosis present or absent on each leg. For further diagnostic characteristics, see Hodgson (1994) and Wang and Feng (2012).

#### Key to species of genus *Coccus* in Korea

**Table:** 

1	Dorsal tubercles absent; legs without tibio-tarsal articulatory scleroses; ventral tubular ducts present on abdomen only	***C. pseudomagnoliarum* (Kuwana)**
–	Dorsal tubercles present; legs with tibio-tarsal articulatory scleroses; ventral tubular ducts present on thorax and abdomen	**2**
2	Ventral tubular ducts of three types (Type I: each with a broad inner ductule; Type II: each with a narrow inner ductule; Type III: each with a filamentous inner ductule) present	***C. malloti* (Takahashi)**
–	Ventral tubular ducts of type I, each with a narrow inner ductule	**3**
3	Antenna 7-segmented; ventral tubular ducts scarce: a small group of 0–3 ducts present between mouthparts and each procoxa; a thin transverse band containing one or two ducts vertically present between mesocoxae; absent on inner submarginal area of abdomen	***C. hesperidum* (Linnaeus)**
–	Antenna 8-segmented; ventral tubular ducts abundant: a large group of 16–20 ducts present between mouthparts and each procoxa; a broad transverse band containing 4–7 ducts present vertically between mesocoxae; present on inner submarginal area of abdomen	***C. ficicola* sp. n.**

### Coccus ficicola sp. n.

Taxon classificationAnimaliaHemipteraCoccidae

http://zoobank.org/1C880C8E-9D53-4418-A87D-EFBBB2227497

Figs 1A–D , 2A–Q

#### Material examined.

**Holotype**: adult female: Korea, Gangnam-gu, Yeoksam-dong, 18.iv.2015, coll. J.Y. Choi, on *Ficus benghalensis* L. (Moraceae). **Paratypes**: same data as holotype, 9♀♀.

#### Diagnosis.

**Figure 1.**
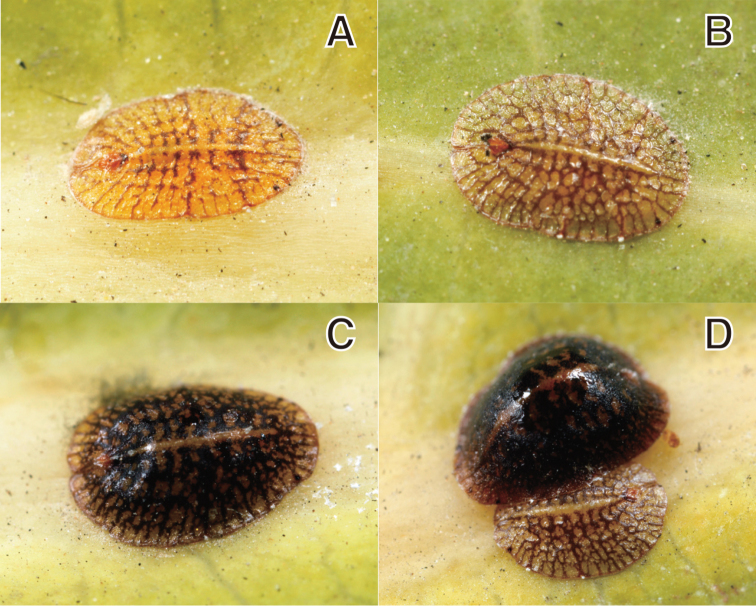
*Coccus ficicola* Choi & Lee sp. n. **A** instar female **B** immature adult female **C** mature adult female **D** mature (upper) and immature (under) adult females.

Adult females in life (Fig. 1A–D) with a reticulated pattern of brown stripes and a longitudinal ridge medially on dorsum; dermal areolations present but small; dorsal tubercles present; dorsal tubular ducts sparse on submarginal area; dorsal setae with bluntly rounded apices; marginal setae usually with simple pointed apices; multilocular disc-pores usually with ten loculi; ventral tubular ducts with a narrow inner ductule, frequent on posterior region of the head, medial area of thorax, and inner submarginal area of abdomen; antennae each with eight segments; legs each with a tibio-tarsal sclerosis on the articulation.

#### Description.

**Adult female. Living appearance** (Fig. 1A–D). Body oval, flattened, or moderately convex. Young adult females yellowish to brownish, with a reticulated pattern of brown stripes except for a longitudinal ridge on mid dorsum. Older adult females becoming more convex and darker. Eggs not seen.

**Figure 2.**
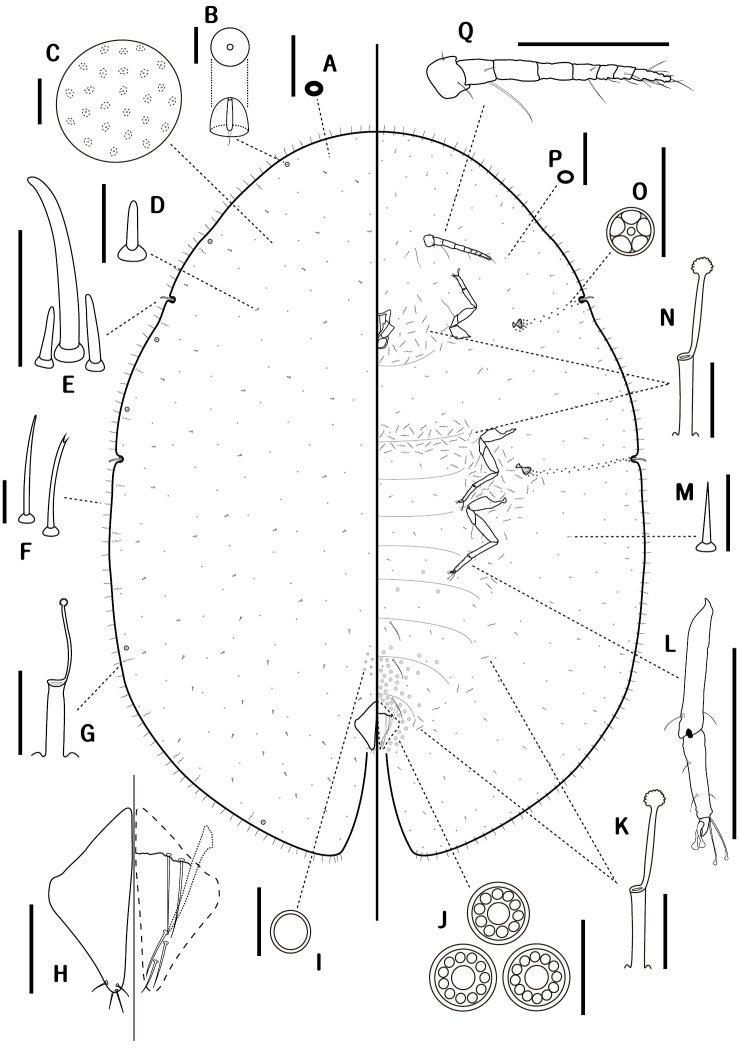
*Coccus ficicola* sp. n., adult female. **A** dorsal microduct **B** dorsal tubercle **C** dermal areolations **D** dorsal seta **E** stigmatic spines **F** marginal setae **G** dorsal tubular duct **H** anal plates **I** preopercular pore **J** multilocular disc-pores **K** ventral tubular duct on abdomen **L** leg **M** ventral seta **N** ventral tubular duct on head and thorax. **O** spiracular pore **P** ventral microduct **Q** antenna. Scale bars: 200 μm (**L, Q**); 100 μm (**H**); 50μm (**C, E**); others = 10μm.

**Slide-mounted material** (Fig. 2A–Q). Body oval, 2.6–3.5 mm long, 2.0–3.6 mm wide, with distinct stigmatic cleft; anal clefts approximately 1/6 of body length.

*Dorsum*. Derm membranous. Dermal areolations oval and small, each with a microduct. Dorsal tubercles normally convex, present on submarginal area, 4–6 in total on each side: two between apex of head and anterior stigmatic cleft, one or two between anterior and posterior stigmatic clefts, and one or two between posterior stigmatic cleft and anal cleft. Dorsal setae cylindrical, short, stout, blunt apically, each 6–9 μm long, moderately distributed on dorsum. Dorsal tubular ducts each with a developed outer ductule and a slender inner ductule with a developed terminal gland, sparsely present on submargin. Dorsal microducts evenly scattered over entire dorsum. Preopercular pores round and small, rather inconspicuous, 6–7 μm wide, set in a small group of approximately 6–15 in front of anal plates. Anal plates quadrate, 190–223 μm long, 160–203 μm wide, usually posterolateral margin slightly longer than anterolateral margin; anterolateral margin 119–144 μm long, posterolateral margin 130–154 μm long; each plate with four apical setae. Ano-genital fold with two pairs of anterior and three pairs of lateral margin setae. Anal ring with six long setae.

*Margin*. Marginal setae spinose, slender, slightly bent, each 16–32 μm long, mostly with simple pointed apices, but sometimes with bifid tips; with 52–59 present between anterior stigmatic clefts; 12–17 laterally present between anterior and posterior stigmatic clefts, 45–53 present between posterior stigmatic cleft and anal clefts. Stigmatic clefts deep, each with three stigmatic spines, median spine 2–3 times as long as lateral spine: medians 48–77 μm long, laterals 14–30 μm long. Eyespots located near margin.

*Venter*. Derm membranous. Multilocular disc-pores 7–8 μm wide, each with 10–12 loculi, mostly with ten loculi, abundant around vulvar area, but less frequent on anterior segments of abdomen. Spiracular pores 4–5 μm wide, each with five loculi, in a narrow band 1–2 pores wide between each spiracle and stigmatic cleft. Ventral tubular ducts of a single type, each 21–28 μm long, with a developed outer ductule and a narrow inner ductule with a flower-shaped terminal gland, approximately 16–20 ducts densely present between mouthparts and procoxa on each side; a broad transverse band containing around 4–7 ducts vertically present between mesocoxae; abundant between each meso- and metacoxa, extending around spiracles; and also sparsely scattered on inner submarginal area between anal plates and each metacoxa. Ventral microducts present on entire venter, especially frequent on submargin. Ventral setae with three pairs of long pregenital setae; two pairs of long setae between antennae; other setae sharply spinose, each 7–14 μm long, moderately distributed over entire venter. Legs well developed, each with a tibio-tarsal articulation and an articulatory sclerosis; total length of each metathoracic leg 560–638 μm long: each coxa 138–163 μm long, trochanter+femur 181–213 μm long, tibia+tarsus 218–237 μm long, claw 17–26 μm long. Tarsal digitules thinner and longer than claw digitules. Spiracles normal, mostly posterior peritreme broader than anterior: anterior peritremes each 38–49 μm wide, posterior peritremes each 45–57 μm wide. Antenna 8-segmented, each 279–339 μm long. Clypeolabral shield 127–138 μm wide.

#### Etymology.

Named after its host plant, *Ficus benghalensis* L.

#### Host plant.

Moraceae: *Ficus benghalensis* L.

#### Comments.

*Coccus ficicola* sp. n. is probably a non-endemic species because it occurs on an imported ornamental plant, *Ficus benghalensis*, which is widely cultivated in tropical areas (Starr et al. 2003). In order to know the exact origin of the new species, further investigations are needed.

#### Morphological comparison of adult females of *Coccus ficicola* sp. n. and its related taxa.

Based on taxonomic articles, such as Gill et al. (1977), Ben-Dov (1981), Avasthi and Shafee (1991), and Lin et al. (2017), we selected ten species morphologically similar to *C. ficicola* sp. n.: *C. capparidis* (Green, 1904), *C. discrepans* (Green, 1904), *C. elatensis* (Ben-Dov, 1981), *C. formicarii* (Green, 1896), *C. gymnospori* (Green, 1908), *C. hesperidum* (Linnaeus, 1758), *C. latioperculatum* (Green, 1922), *C. moestus* (De Lotto, 1959), *C. praetermissus* Lin & Tanaka, 2017, and *C. sulawesicus* Gavrilov, 2013. The morphological characters of adult females of *Coccus ficicola* and the ten species are summarized in Table 1.

**Table 1.** Comparison of morphological characters of adult females of *Coccus ficicola* sp. n. and its related taxa.

Species	Dorsal tubercles	Dorsal tubular ducts	Dorsal setae	Preopercular pores	Marginal setae	Antenna	Pregenital setae	Tibio-tarsal sclerosis	Ventral tubular ducts	Reference
*C. ficicola* sp. n.	Present	Present	Bluntly rounded	Present	Pointed or frayed	Eight segments	Three pairs	Present	Present on medial area of head, pro-, and mesothorax; submarginal area of abdomen	This study
*C. capparidis*	Present	Absent	Bluntly rounded	Present	Pointed or frayed	Six or seven segments	One or two pairs	Absent	Present on submarginal area of abdomen	Williams and Watson 1990
*C. discrepans*	Present	Absent	Sharply pointed or bluntly rounded	absent	Pointed or frayed	Seven segments	Three pairs	Present	Present on medial area of mesothorax	Avasthi and Shafee 1991; Tao et al. 1983
*C. elatensis*	Present	Present	Bluntly rounded	Present	Pointed or frayed	Eight segments	Four pairs	Present	Present on medial area of mesothorax	Ben-Dov 1981
*C. formicarii*	Absent	Absent	Sharply pointed (setose)	Present	Pointed (setose)	Seven or eight segments	Three pairs	Absent	Present on medial area of head, pro-, and mesothorax	Hodgson 1994
*C. gymnospori*	Present	Present	Bluntly rounded	Present	Pointed or frayed	Eight segments	Three pairs	Present	Present on medial area of head and mesothorax	Ben-Dov 1981
*C. hesperidum*	Present	Present or absent	Sharply pointed	Present	Pointed or frayed	Seven segments	Three pairs	Present	Present on medial area of pro- and mesothorax; laterad to genital opening	Hodgson 1994; Lin et al. 2017
*C. latioperculatum*	Absent	Absent	Bluntly rounded	Present	Frayed	Seven segments	Two pairs	Present	Present on medial area of pro- and mesothorax	Avasthi and Shafee 1991
*C. moestus*	Present	Present	Bluntly rounded	Present	Frayed	Seven or eight segments	Three pairs	Present	Present on medial area of mesothorax	Gill et al. 1977
*C. praetermissus*	Present	Present	Bluntly rounded	Present	Pointed or frayed	Seven segments	Three pairs	Present	Present on medial area of mesothorax	Lin et al. 2017
*C. sulawesicus*	Absent	Absent	Sharply pointed	Present	Pointed or frayed	Seven or eight segments	Three pairs	Present	Present on medial area of meso- and metathorax	Gavrilov-Zimin 2013

In the morphological comparison, *Coccus ficicola* shows a new combination of morphological characters; in particular, the distributional pattern of ventral tubular ducts of the species reveals uniqueness among the nine morphological characters. *Coccus ficicola* is most closely related to *C. gymnospori* (Green), in having (i) dorsal tubercles, (ii) dorsal tubular ducts on submarginal area, (iii) dorsal setae with bluntly rounded apices, (iv) preopercular pores, (v) marginal setae with pointed or frayed apices, (vi) antenna with eight segments, (vii) three pairs of pregenital setae, and (viii) tibio-tarsal sclerosis. However, *C. ficicola* differs from *C. gymnospori* in having the following combination of character states (character states of *C. gymnospori* in parenthesis): (i) ventral tubular ducts abundant, 16 to 20 ducts present between mouthparts and each procoxa (few, only 3 or 4 ducts); a broad transverse band containing 4–7 ducts vertically between metacoxae (thin, containing one or two ducts); and present on inner submarginal area of abdomen (entirely absent), and (ii) multilocular disc-pores extending further anteriorly (restricted to preceding two abdominal segments) (Ben-Dov 1981; Avasthi and Shafee 1989).

Although the African species, *C. africanus* (Newstead) and *C. alpinus* De Lotto, are not included in the list of related taxa for morphological comparison, *C. ficicola* is similar to both species in having abundant ventral tubular ducts. However, *C. ficicola* does not have continuous ventral tubular ducts between the metacoxae, whereas both African species have this character state (De Lotto 1957; De Lotto 1960; Granara de Willink et al. 2010).

### Coccus hesperidum

Taxon classificationAnimaliaHemipteraCoccidae

(Linnaeus, 1758)

Fig. 3A–F

Coccus hesperidum Linnaeus, 1758: 455.

#### Material examined.

Adult female: Daehak-dong, Gwanak-gu, Seoul, 09.iv.2014, coll. J.Y. Choi, on Orchidaceae sp., 5♀♀; Sinhyo-dong, Seogwipo-si, Jeju-do, 14.ix.2014, coll. J.Y. Choi, on *Asplenium antiquum* Makino (Aspleniaceae), 5♀♀; Sinbuk-eup, Chuncheon-si, Gangwon-do, 31.v.2015, coll. J.Y. Choi, on *Heteropanax fragrans* (Roxb.) (Araliaceae), 5♀♀; Geumam-dong, Deokjin-gu, Jeonju-si, Jeollabuk-do, 06.vi.2015, on same host, 5♀♀; Songhyeon-dong, Andong-si, Gyeongsangbuk-do, 07.vi.2015, on same host, 5♀♀; Guseo-dong, Geumjeong-gu, Busan, 07.vi.2015, on *Ficus benghalensis* L., 5♀♀.

#### Diagnosis.

**Figure 3.**
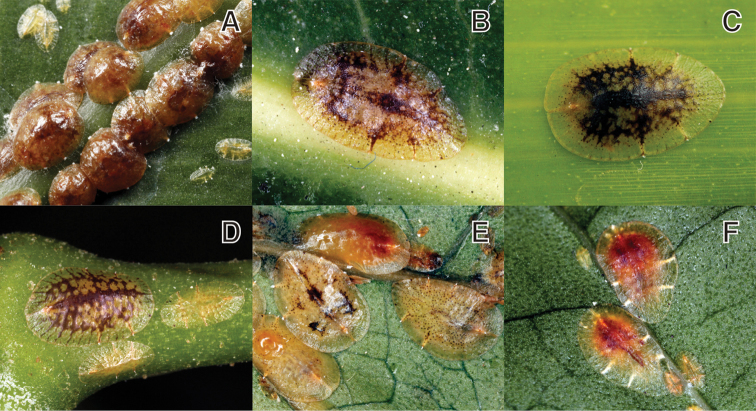
*Coccus hesperidum* (Linnaeus, 1758). **A** population on *Asplenium antiquum* **B** adult female on *Ficus benghalensis* **C** adult female on Orchidaceae sp. **D, E, F** populations on *Heteropanax fragrans*.

Adult females in life (Fig. 3A–F) highly variable in body color and pigment pattern, but usually dorsum pale yellowish to brownish, with black or brown spots; dermal areolations present; dorsal tubercles present; dorsal tubular ducts present or absent; dorsal setae with sharply pointed apices; marginal setae usually with pointed, bifid or fimbriate apices; multilocular disc-pores usually with ten loculi; ventral tubular ducts with a narrow inner ductule, few present around meso- and procoxa, and anal plates; antennae each 7-segmented; legs each with a tibio-tarsal articulatory sclerosis.

**Host plant**. Recorded from 346 genera in 121 families (García-Morales et al. 2016). For Korean records, see Paik (1978).

#### Distribution.

Known from all zoogeographical regions (García-Morales et al. 2016).

### Coccus malloti

Taxon classificationAnimaliaHemipteraCoccidae

(Takahashi, 1956)

Figs 4A–B , 5A–Q

Pulvinaria malloti Takahashi, 1956: 25.

#### Material examined.

Adult female: Jeollanam-do, Gwangyang-si, Ongnyong-myeon, Chusan-ri, 28.v.2015, coll. J.Y. Choi, on *Ilex cornuta* Lindl. (Aquifoliaceae), 9♀♀; Jeju-do, Seogwipo-si, Andeok-myeon, Gamsan-ri, 27.iv.2016, coll. J.Y. Choi, on *Aphananthe aspera* (Thunb.) (Cannabaceae), 1♀.

#### Diagnosis.

**Figure 4.**
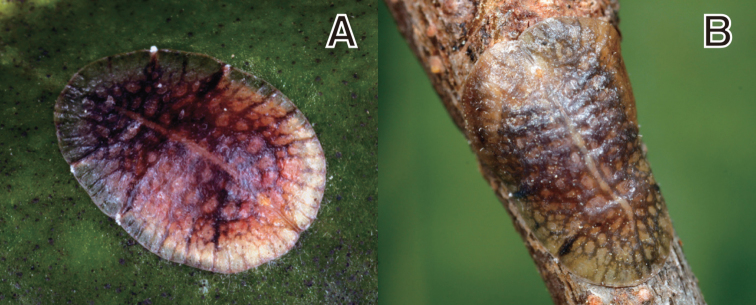
*Coccus malloti* (Takahashi, 1956). **A** adult female on *Ilex cornuta* **B** adult female on *Aphananthe aspera*.

Adult females in life (Fig. 4A–B) with a reticulated pattern of black stripes and a longitudinal band medially on dorsum; dermal areolations present but small; dorsal tubercles present; dorsal tubular ducts absent; dorsal setae sharply spinose; marginal setae mostly with simple pointed apices; multilocular disc-pores usually with ten loculi; ventral tubular ducts of three types: Type I with a broad inner ductule, densely present on posterior medial area of head; frequent on anterior medial area of prothorax, extending to inner submarginal area of thorax; and also sparsely scattered on inner submarginal area of abdomen; Type II with a narrow inner ductule and a large flower-shaped terminal gland, rarely present on inner submarginal area and posterior medial area of abdomen; Type III with a long filamentous inner ductule and a quite small terminal gland, moderately present on submarginal area between anal clefts and each posterior spiracular furrow; all types of ventral tubular ducts absent on medial area of meso-, metathorax and anterior abdomen, and submarginal area of head; antennae each eight segments; legs each with a tibio-tarsal sclerosis on the articulation.

#### Redescription.

**Adult female. Living appearance** (Figs 4A–B). Body elongate oval, flattened, or slightly convex. Young adult females yellowish to dark brownish, with a reticulated pattern of brown or black stripes, getting darker at maturity. Eggs reddish in color, stored beneath venter.

**Figure 5.**
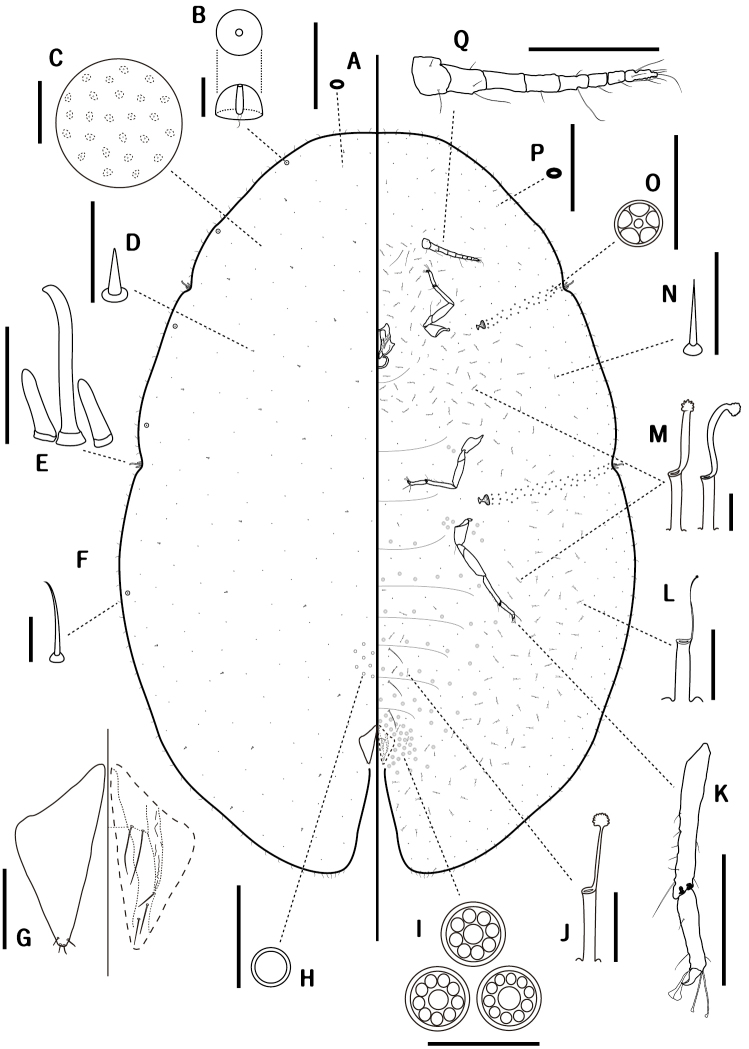
*Coccus malloti* (Takahashi, 1956), adult female. **A** dorsal microduct **B** dorsal tubercle **C** dermal areolations **D** dorsal seta **E** stigmatic spines **F** marginal seta **G** anal plates **H** preopercular pore **I** multilocular disc-pores **J** ventral tubular duct (Type II) **K** leg **L** ventral tubular duct (Type III) **M** ventral tubular ducts (Type I) **N** ventral seta **O** spiracular pore **P** ventral microduct **Q** antenna. Scale bars: 200 μm (**K, Q**); 100μm (**G**); 50 μm (**C, E**); others = 10 μm.

**Slide-mounted material** (Fig. 5A–Q). Body elongate oval, 3.6–4.8 mm long, 2.2–3.3 mm wide, with shallow to deep stigmatic cleft; anal clefts approximately 1/6 of body length.

*Dorsum*. Derm membranous. Dermal areolations oval and small, each with a microduct. Dorsal tubercles normally convex, present on submarginal area, 1–5 in total on each side: one or two between apex of head and anterior stigmatic cleft, zero to two between anterior and posterior stigmatic clefts, and zero or one between posterior stigmatic cleft and anal cleft. Dorsal setae sharply spinose, short, stout, each 6–9 μm long, moderately distributed on dorsum. Dorsal tubular ducts absent. Dorsal microducts evenly scattered over entire dorsum. Preopercular pores round and small, 3–5 μm wide, set in a small group of approximately 8 in front of anal plates. Anal plates quadrate, 217–249 μm long, 169–198 μm wide, each with slightly concaved posterolateral margin, usually posterolateral margin quite longer than anterolateral margin; anterolateral margin 123–143 μm long, posterolateral margin 150–169 μm long; each plate with four apical setae. Ano-genital fold with two pairs of anterior and three pairs of lateral margin setae. Anal ring with six long setae.

*Margin*. Marginal setae spinose, straight or slightly bent, each 14–22 μm long, mostly with simple pointed apices; with 30–43 present between anterior stigmatic clefts; 13–17 laterally present between anterior and posterior stigmatic clefts, 22–38 present between posterior stigmatic cleft and anal clefts. Stigmatic clefts shallow to deep, each with three stigmatic spines, median spine nearly twice as long as lateral spine: medians 60–75μm long, laterals 24–38 μm long. Eyespots located near margin.

***Venter.*** Derm membranous. Multilocular disc-pores 6–7 μm wide, each with 8–10 loculi, mostly with ten loculi, abundant around vulvar area; one or two transverse rows on each abdominal segments; and also small groups present laterad of each metacoxa and mesocoxa, but not observed around procoxa. Spiracular pores 4–5 μm wide, each with five loculi, in a narrow band 2–4 pores wide between each spiracle and stigmatic cleft. Ventral tubular ducts of three types: Type I each with 33–39 μm long, with a developed outer ductule and a moderately broad inner ductule, straight or slightly curved, with a flower-shaped terminal gland, densely present on posterior medial area of head; frequent on anterior medial area of prothorax, extending to inner submarginal area of thorax; and also sparsely scattered on inner submarginal area of abdomen; Type II each with 16–24 μm long, with a narrow inner ductule and a large flower-shaped terminal gland, rarely present on inner submarginal area and posterior medial area of abdomen; Type III each with 15–24 μm long, with a long filamentous inner ductule and a quite small terminal gland, moderately present on submarginal area between anal clefts and each posterior spiracular furrow; all types of ventral tubular ducts absent on medial area of meso- and metathorax and anterior abdomen, and submarginal area of head. Ventral microducts present on entire venter, especially frequent on submargin. Ventral setae with three pairs of long pregenital setae; approximately three or four pairs of long and short setae between antennae; other setae sharply spinose, each 5–10 μm long, sparsely distributed over entire venter. Legs well developed, each with a tibio-tarsal articulation and an articulatory sclerosis; total length of each metathoracic leg 614–769 μm long: each coxa 141–189 μm long, trochanter+femer 209–257 μm long, tibia+tarsus 243–311 μm long, claw 17–24 μm long. Tarsal digitules thinner and longer than claw digitules. Spiracles normal, mostly posterior peritreme broader than anterior: anterior peritremes each 37–52 μm wide, posterior peritremes each 45–61 μm wide. Antenna 8-segmented, each 339–399 μm long. Clypeolabral shield 138–157 μm wide.

#### Host plant.

Recorded from six genera in six families (García-Morales et al. 2016). In Korea, it was found on *Aphananthe aspera* (Cannabaceae) and *Ilex cornuta* (Aquifoliaceae).

#### Distribution.

Only known from Japan (Takahashi 1956); first record for Korea.

#### Comments.

The above description based on Korean specimens agrees well with that of Takahashi (1956), except that variation in the number of dorsal tubercles and marginal setae, and exact distributions of each type of ventral tubular ducts are newly provided in this study. *Coccus malloti* probably has intermediate morphological characters between the tribes Coccini and Pulvinariini. However, the woolly test, known as ovisac and one of the typical characters of the Pulvinariini, is not observed in the species. In addition, some slide-mounted specimens of *C. malloti* contain eggs and nymphs in their body, which indirectly indicates that they would not produce an ovisac for oviposition. Although *Coccus malloti* would be retained in the tribe Coccini, it needs to be reviewed with its type materials to clarify the exact generic position of the species.

### Coccus pseudomagnoliarum

Taxon classificationAnimaliaHemipteraCoccidae

(Kuwana, 1914)

Fig. 6A–B

Lecanium (Eulecanium) pseudomagnoliarum Kuwana, 1914: 7.

#### Material examined.

Adult female: Sujeong-dong, Yeosu-si, Jeollanam-do, 27.v.2015, coll. J.Y. Choi, on *Celtis* sp. (Cannabaceae), 10♀♀.

#### Diagnosis.

**Figure 6.**
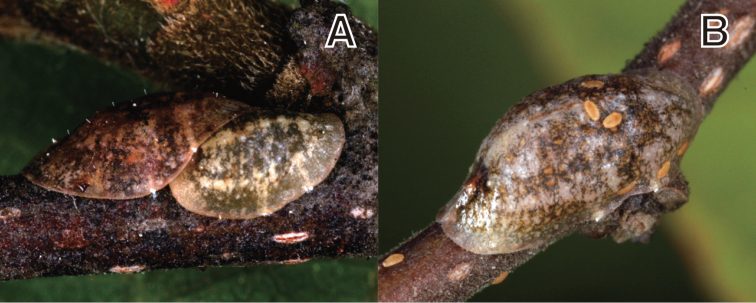
*Coccus pseudomagnoliarum* (Kuwana, 1914). **A** immature adult females **B** mature adult female and 1^st^ instars.

Adult females in life (Fig. 6A–B) greenish or greyish, with light or dark yellow mottling; dermal areolations present; dorsal tubercles absent; dorsal tubular ducts absent; dorsal setae with sharply pointed apices; marginal setae with simple pointed or spatulate apices; multilocular disc-pores usually with 6–10 loculi; ventral tubular ducts with a narrow inner ductule, few present on submaginal area of posterior abdomen; antennae each 8-segmented; legs without tibio-tarsal articulatory scleroses.

#### Host plant.

Recorded from six genera in five families (García-Morales et al. 2016). In Korea, it has been recorded from the following plants: *Celtis willdenowiana* (Cannabaceae), *Citrus* sp., *Phellodendron amurense*, *Poncirus trifoliata* (Rutaceae), *Clerodendron trichotomum* (Lamiaceae), and *Zelkova serrata* (Ulmaceae) (Paik 1978).

#### Distribution.

Mainly known from Palearctic Region including Australia, Europe, Iran, Israel, Japan, Russia, Korea, and USA (García-Morales et al. 2016).

## Discussion

Recently, Lin et al. (2017) described *Coccus praetermissus* Lin & Tanaka, which could be confused with a cosmopolitan species, *Coccus hesperidum* Linnaeus, 1758, based on morphological and molecular evidences. They pointed out that a morphological difference exists between the adult females of two genetically distinct species, the shape of dorsal setae, although molecular data (COI) should be used for exact identification. The adult female of *Coccus praetermissus* has dorsal setae with bluntly rounded apices, whereas those of *C. hesperidum* have sharply pointed tips. *Coccus ficicola* sp. n. is close to *C. praetermissus* in having the former type of dorsal setae, but differs in the distributional pattern of ventral tubular ducts, which is a reliable and constant character in each species within the genus *Coccus*. The ventral tubular ducts of *Coccus ficicola* are present on medial area of head, pro- and mesothorax, and submarginal area of abdomen, whereas *C. praetermissus* has the structures on medial area of mesothorax only.

Under the morphological comparison with congeners, we conclude that *Coccus ficicola* sp. n. is a distinct species which is a morphologically differentiated lineage. The distinctive pattern of ventral tubular ducts seems to be an autapomorphic feature of *Coccus ficicola* because it shows uniqueness in the comparison of morphological characters. In order to clarify the phylogenetic relationships of a new species within the genus *Coccus*, molecular analysis employing mitochondrial and nuclear loci are required.

## Supplementary Material

XML Treatment for Coccus

XML Treatment for Coccus ficicola

XML Treatment for Coccus hesperidum

XML Treatment for Coccus malloti

XML Treatment for Coccus pseudomagnoliarum
